# Predictive Accuracy of Ultrasound Biometry and Maternal Factors in Identifying Large-for-Gestational-Age Neonates at 30–34 Weeks

**DOI:** 10.3390/diagnostics16020187

**Published:** 2026-01-07

**Authors:** Vasileios Bais, Antigoni Tranidou, Antonios Siargkas, Sofoklis Stavros, Anastasios Potiris, Dimos Sioutis, Chryssi Christodoulaki, Apostolos Athanasiadis, Apostolos Mamopoulos, Ioannis Tsakiridis, Themistoklis Dagklis

**Affiliations:** 1 Department of Obstetrics and Gynaecology, School of Medicine, Faculty of Health Sciences, University of Ioannina, 45332 Ioannina, Greece; 2 Third Department of Obstetrics and Gynaecology, School of Medicine, Faculty of Health Sciences, Aristotle University of Thessaloniki, 54124 Thessaloniki, Greece; 3 Third Department of Obstetrics and Gynecology, University Hospital “ATTIKON”, Medical School, National and Kapodistrian University of Athens, 17237 Athens, Greece

**Keywords:** large-for-gestational-age, fetal biometry, uterine artery Doppler, prediction model, macrosomia

## Abstract

**Background/Objectives:** To construct and compare multivariable prediction models for the early prediction of large-for-gestational-age (LGA) neonates, using ultrasound biometry and maternal characteristics. **Methods:** This retrospective cohort study analyzed data from singleton pregnancies that underwent routine ultrasound examinations at 30^+0^–34^+0^ weeks of gestation. Ultrasound parameters included fetal abdominal circumference (AC), head circumference (HC), femur length (FL), HC-to-AC ratio, mean uterine artery pulsatility index (mUtA-PI), and presence of polyhydramnios. LGA neonates were defined as those having a birthweight > 90th percentile. Logistic regression was used to evaluate associations between ultrasound markers and LGA after adjusting for the following maternal and pregnancy-related covariates: maternal age, body mass index, parity, gestational diabetes mellitus (GDM), pre-existing diabetes, previous cesarean section (PCS), assisted reproductive technology (ART) use, smoking, hypothyroidism, and chronic hypertension. Associations were expressed as adjusted odds ratios (aORs) with 95% confidence intervals (CIs). Three prognostic models were developed utilizing the following predictors: (i) biometric ultrasound measurements including AC, HC-to-AC ratio, FL, UtA-PI, and polyhydramnios (Model 1), (ii) a combination of biometric ultrasound measurements and clinical–maternal data (Model 2), and (iii) only the estimated fetal weight (EFW) (Model 3). **Results:** In total, 3808 singleton pregnancies were included in the analyses. The multivariable analysis revealed that AC (aOR 1.07, 95% CI [1.06, 1.08]), HC to AC (aOR 1.01, 95% CI [1.006, 1.01]), FL (aOR 1.01, 95% CI [1.009, 1.01]), and the presence of polyhydramnios (aOR 4.97, 95% CI [0.7, 58.8]) were associated with an increased risk of LGA, while a higher mUtA-PI was associated with a reduced risk (aOR 0.98, 95% CI [0.98, 0.99]). Maternal parameters, such as GDM, pre-existing diabetes, elevated pre-pregnancy BMI, absence of uterine artery notching, mUtA-PI, and multiparity, were significantly higher in the LGA group. Both models 1 and 2 showed similar performance (AUCs: 84.7% and 85.3%, respectively) and outperformed model 3 (AUC: 77.5%). Bootstrap and temporal validation indicated minimal overfitting and stable model performance, while decision curve analysis supported potential clinical utility. **Conclusions:** Models using biometric and Doppler ultrasound at 30–34 weeks demonstrated good discriminative ability for predicting LGA neonates, with an AUC up to 84.7%. Adding maternal characteristics did not significantly improve performance, while the biometric model performed better than EFW alone. Sensitivity at conventional thresholds was low but increased substantially when lower probability cut-offs were applied, illustrating the model’s threshold-dependent flexibility for early risk stratification in different clinical screening needs. Although decision curve analysis was performed to explore potential clinical utility, external validation and prospective assessment in clinical settings are still needed to confirm generalizability and to determine optimal decision thresholds for clinical application.

## 1. Introduction

Antenatal identification of large-for-gestational-age (LGA) neonates is important for optimizing prenatal care, planning delivery, and mitigating potential complications associated with fetal overgrowth [1]. LGA, defined as birthweight > 90th percentile for gestational age, is associated with an increased risk of labor complications, including shoulder dystocia, birth trauma, and neonatal intensive care unit admissions [1,2,3]. Moreover, LGA infants face increased risk of adverse metabolic outcomes later in life, such as obesity, hypertension, and type 2 diabetes [4,5]. In cases where LGA is detected, underlying maternal and fetal conditions should be excluded. These conditions include gestational diabetes mellitus (GDM) and congenital overgrowth syndromes, specifically Beckwith–Wiedemann, Pallister–Killian, Sotos, Perlman, and Simpson–Golabi–Behmel syndromes [6].

One strategy to mitigate these risks is early induction of labor. Evidence from a large randomized controlled trial demonstrated that induction at 37^+0^–39^+0^ weeks of gestation reduced the incidence of severe shoulder dystocia by 68%, suggesting a potential clinical benefit [7]. However, despite these findings, national and international guidelines do not currently recommend routine early induction of labor for suspected LGA unless additional risk factors, such as maternal diabetes, are present [8,9]. Furthermore, a recent meta-analysis investigating the timing of induction in suspected macrosomia showed that gestational age at induction has a decisive role in perinatal adverse outcomes, further highlighting the need for effective strategies to identify and appropriately manage these pregnancies [10].

There is no consensus on the routine sonographic evaluation of low-risk pregnancies in the third trimester [11]. Accumulating data suggests that third-trimester ultrasonography may offer several advantages, including the identification of small-for-gestational-age (SGA) and LGA fetuses [12], assessment of placental position, fetal presentation, and detection of late-onset anomalies [13]. Another point of dispute is the timing of a routine third-trimester ultrasound. Traditionally, this was offered at 30–34 weeks of gestation. However, it was recently advocated that in low-risk pregnancies, a late third-trimester scan, at 35–37 weeks, may be more useful in identifying growth abnormalities and allow for more effective decision making on management [14]. The 30–34-week window was chosen in this study to investigate whether earlier prediction could allow proactive planning. Nonetheless, this earlier window presents a greater challenge for predicting LGA neonates, as most excessive fetal growth occurs closer to term. Fetal growth is traditionally evaluated using ultrasound measurements, but this approach is often inaccurate in predicting LGA, particularly when used in isolation and early in the third trimester [15]. Standard third-trimester estimated fetal weight assessments often lack the necessary sensitivity and specificity, which can result in both false-positive and false-negative diagnoses, potentially leading to unnecessary interventions or missed opportunities for appropriate management [15]. To overcome this limitation, various multivariable prediction models have been developed to improve the sensitivity of ultrasound alone, incorporating either additional ultrasound parameters or maternal factors [1]. Given that maternal factors—such as pre-pregnancy body mass index (BMI), GDM, and parity— significantly influence fetal growth trajectories, it is reasonable to expect that integrating these variables into predictive models alongside ultrasound biometry would enhance predictive accuracy [16,17,18]. However, a recent systematic review that evaluated all multivariable prediction models for detecting LGA concluded that most models had similar or lower area under the curve (AUC) values compared to ultrasound alone, and that none of the models are ready for clinical implementation yet [1].

Therefore, the primary objective of this study was to evaluate if a model using fetal biometric measurements [abdominal circumference (AC), femur length (FL), head circumference (HC)-to-AC ratio], uterine artery Doppler parameters [mean uterine artery pulsatility index (mUtA-PI)], and the presence of polyhydramnios has superior predictive performance for LGA neonates compared to estimated fetal weight (EFW) alone, based on a single examination at 30–34 weeks of gestation. We also aimed to determine if the addition of maternal clinical factors could improve this model’s accuracy. The aim is to build a more accurate prediction model that helps clinicians focus fetal monitoring on pregnancies with the greatest risk.

## 2. Materials and Methods

### 2.1. Study Details and Population Characteristics

We conducted a retrospective cohort study using data from singleton pregnancies that underwent routine ultrasound examinations between 30^+0^ and 34^+0^ weeks of gestation at the 3rd Department of Obstetrics and Gynecology, School of Medicine, Faculty of Health Sciences, Aristotle University of Thessaloniki, Greece. All eligible women who came to the clinic during the data collection period were consecutively included in the study. Data collection took place between February 2013 and February 2024. Data were obtained from dedicated medical records, encompassing demographic information, medical history, and ultrasound measurements. Based on the national guidelines and the local clinical protocol, all participants were offered routine ultrasound examinations at 11^+0^–13^+6^ weeks, 20^+0^–23^+6^ weeks, and at 30^+0^–34^+0^ weeks. Although later third-trimester scans may improve predictive accuracy for LGA detection, the 30–34-week window was selected to explore earlier identification, allowing for potential intervention planning. All examinations were performed by fetal medicine specialists, accredited by the Fetal Medicine Foundation, UK, using the Voluson E8 ultrasound system (GE HealthCare, Chicago, IL, USA) with the GE RAB4-8-D transducer (GE HealthCare, Chicago, IL, USA). The ultrasound parameters assessed included HC, AC, FL, HC-to-AC ratio, presence of polyhydramnios defined as the deepest pool of amniotic fluid > 8 cm, and mUtA-PI. In addition, uterine artery notching was recorded and categorized as absent, bilateral, left-sided, or right-sided. Fetal growth was assessed using the Hadlock growth curves as reference standards, with no adjustment for fetal sex in the calculation of centiles. Furthermore, all women underwent routine screening for GDM between 26^+0^ and 27^+6^ weeks of gestation, according to criteria based on the results of the HAPO study [19]. According to this protocol, GDM was defined as a fasting plasma glucose of 5.1 mmol/L or more, a 1 h value of 10.0 mmol/L or more, or a 2 h value of 8.5 mmol/L or more after a 75 g oral glucose load. Maternal factors, such as previous cesarean section (PCS), GDM diagnosed according to HAPO study criteria [19], conception via assisted reproductive technology (ART), parity, pre-pregnancy BMI, maternal age, smoking during pregnancy, presence of hypothyroidism, pre-existing diabetes (type I or II), and chronic hypertension, were also noted. Of note, parity was analyzed as a dichotomous variable, while BMI was treated as a continuous variable.

Eligibility criteria were as follows: (i) maternal age ≥ 18 years old, (ii) previous scans at 11^+0^–13^+6^ weeks and 20^+0^–23^+6^ weeks, (iii) singletons with no detectable congenital anomalies, (iv) availability of complete records for key ultrasound measurements (AC, HC, FL, and mUtA-PI) and relevant maternal factors, and (v) deliveries after 33^+1^ weeks with known perinatal outcomes.

LGA was defined as birth weight > 90th percentile for gestational age based on the Hadlock growth charts, which the recent literature suggests perform comparably to the INTERGROWTH charts [20].

This study was part of a larger prospective cohort conducted in accordance with the principles of the Declaration of Helsinki [21]. Ethical approval was granted by the Bioethics Committee of the Aristotle University of Thessaloniki, Greece (approval No. 6.231/29 July 2020) before the start of the study. All participants provided written informed consent prior to enrollment, and no financial or other incentives were offered for participation.

### 2.2. Statistical Analysis

The HC, AC, FL, HC to AC, and mUtA-PI centiles were calculated using the mathematical models reported by Snijders et al. [22], Gómez et al. [23], and Sotiriadis et al. [24], respectively. To describe the characteristics of the study’s population, the Shapiro–Wilk test was applied to assess the normality of the continuous variables, while the F-test was used to check the equality of variances. Depending on data distribution, hypothesis testing was performed using the t-test for normally distributed data, and the Wilcoxon or Mann–Whitney tests for non-parametric data. For binary variables, Fisher’s exact test was used, particularly in cases with small sample sizes. Three logistic regression models were constructed:

Biometric Ultrasound Measurements Model (Model 1): Included AC, FL, HC to AC, mUtA-PI, and presence/absence of polyhydramnios as predictors.

Biometric and Clinical Model (Model 2): Included the same variables as Model 1 plus maternal factors (maternal age, BMI, parity, GDM, pre-existing diabetes, PCS, ART use, smoking, hypothyroidism, and chronic hypertension).

EFW Model (Model 3): Used the EFW as the only predictor, estimated according to the Hadlock 4 (HC, AC, and FL) formula [25]. EFW was derived from AC, HC, and FL. It was analyzed separately to compare the predictive value of raw biometric inputs and Doppler measures versus the composite EFW formula.

The study population was stratified into two groups: pregnancies complicated by GDM or pre-existing diabetes, and those without these conditions. The same three logistic regression models were then developed separately within each group. Sensitivity, specificity, positive predictive value (PPV), and negative predictive value (NPV) were calculated to assess predictive performance. To further evaluate the model’s discrimination ability, a receiver operating characteristic (ROC) curve was plotted, and the AUC was calculated along with its 95% confidence interval (CI). A *p*-value of less than 0.05 was considered statistically significant. All analyses were conducted using the R software (version 4.2.1).

Missing data were assessed for all predictors included in the analysis, and the extent of missingness is presented in Supplementary Table S1. Overall, the dataset was highly complete. Only pre-pregnancy BMI showed missing values (89/3808, 2.34%), while all other variables had 0% missingness. Because the proportion of missing data was small and limited to a single predictor, complete-case analysis was performed. This approach is consistent with methodological recommendations indicating that complete-case analysis is unlikely to introduce meaningful bias when the proportion of missingness is approximately < 5% [26]. Furthermore, TRIPOD does not prescribe a specific threshold at which imputation must be applied but requires transparent reporting of missing data and the chosen handling strategy, which is fulfilled here [27]. Given the minimal level of missingness, additional imputation was not considered necessary.

Given the retrospective design, no formal a priori sample size calculation was performed. Sample adequacy was evaluated according to the number of outcome events per predictor, which exceeded the commonly recommended minimum of 10 events per predictor for reliable model development [28].

Internal validation was performed using bootstrap resampling with optimism correction. For each of the three prediction models, 500 bootstrap samples were drawn with replacement from the original dataset (*n* = 3808; 431 LGA cases, 3377 non-LGA cases). In each bootstrap iteration, the model was fitted on the bootstrap sample and performance was evaluated on both the bootstrap sample (apparent performance) and the original dataset (test performance). Optimism was calculated as the difference between apparent and test performance for each metric. The mean optimism across all 500 bootstrap iterations was then subtracted from the apparent performance on the original dataset to obtain optimism-corrected estimates. Bootstrap validation was performed to evaluate model performance across several metrics. Discrimination was assessed using the area under the receiver operating characteristic curve (AUC), and overall predictive accuracy was measured with the Brier score. Model calibration was examined through the calibration intercept and slope. In addition, sensitivity, specificity, positive predictive value (PPV), and negative predictive value (NPV) were calculated using a probability threshold of 0.5. All bootstrap procedures and statistical analyses were carried out in R version 4.3 (R Foundation for Statistical Computing, Vienna, Austria).

Temporal validation was performed using chronological stratification of the dataset. The cohort was divided into an earlier subset used for model development (*n* = 2599, including 291 LGA cases) and a later subset used for testing (*n* = 1118, including 136 LGA cases). Prediction models were developed using the earlier subset and subsequently evaluated in the later subset. Model discrimination was assessed using the AUC, and differences in the AUC between the two temporal subsets were calculated to quantify changes in performance over time.

Decision curve analysis was performed to evaluate the potential clinical utility of the prediction models across a range of threshold probabilities. Net benefit was calculated for models 1, 2, and 3 and compared with default strategies of treating all pregnancies as high risk and treating none. Net benefit represents the balance between true positives and false positives at different risk thresholds, allowing for the assessment of whether decision making based on the models would improve clinical outcomes.

To assess potential circularity between fetal biometric predictors and the definition of LGA, sensitivity analysis was performed using an alternative representation of fetal biometry. In the main analysis, biometric variables were expressed as gestational age-adjusted centiles. In the sensitivity analysis, centile-based predictors were replaced with raw biometric measurements and gestational age was included explicitly as a covariate. Model discrimination was compared between approaches using the AUC.

## 3. Results

**Figure 1:**
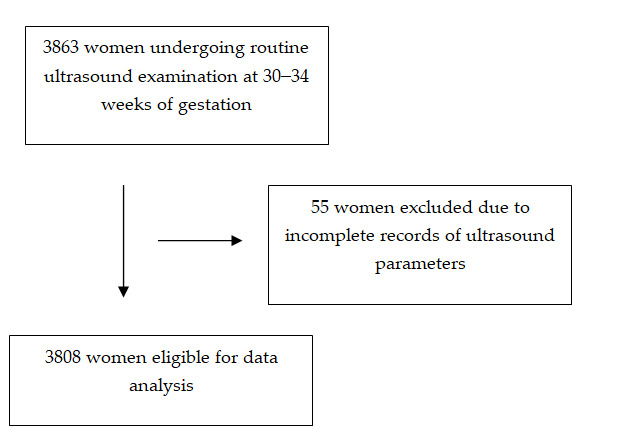
Flowchart of the investigated population selection process.

The study population consisted of 3863 women undergoing routine ultrasound examination at 30^+0^–34^+0^ weeks of gestation with singleton pregnancies and structurally normal fetuses. Of these, 55 women were excluded due to incomplete records of ultrasound parameters. Thus, 3808 women were eligible for the data analysis (Figure 1).

Of the recruited women, 431 (11.32%) delivered LGA neonates, while 722 (18.96%) delivered SGA neonates. The general characteristics of the population are presented in Table 1. In the unadjusted analysis, women who delivered an LGA neonate had a significantly higher pre-pregnancy BMI and a higher prevalence of PCS, GDM, multiparity, and pre-existing diabetes mellitus. Fetal biometrics percentiles (AC, HC, and FL) were also significantly higher in the LGA group, although the HC-to-AC ratio was lower. Additionally, the absence of a uterine artery notch and the presence of polyhydramnios were significantly more common in the LGA group (Table 1).

In the adjusted analysis, AC centile (aOR 1.06, 95% CI [1.06, 1.07]), HC/AC centile (aOR 1.01, 95% CI [1.006, 1.01]), and FL centile (aOR 1.01, 95% CI [1.009, 1.01]) remained significantly associated with LGA. The mUtA-PI centile was associated with a reduced likelihood of LGA (aOR 0.98, 95% CI [0.98, 0.99]). Polyhydramnios showed a positive but non-significant association with LGA (aOR 4.97, 95% CI [0.7–58.8], *p*-value = 0.14) (Table 2).

The prognostic performances of Models 1 and 2 did not differ significantly. For Model 1, sensitivity was 23.4%, specificity was 98.2%, and the AUC was 84.7%. For Model 2, sensitivity was 23.4%, specificity was 98.2%, and the AUC was 85.3%. In comparison, the EFW model (Model 3) achieved inferior performance, with a sensitivity of 7.4%, specificity of 99.2%, and an AUC of 77.5% (Table 3). The post hoc power of the study was high for all models and the systematic error of the ROC curves approached zero, indicating sufficient sample size and minimal bias across all possible thresholds.

Performance metrics were also calculated at multiple decision thresholds for all three models (Supplementary Tables S2–S4). At the conventional 0.5 threshold, the sensitivities for Model 1, Model 2, and Model 3 were 23.4%, 23.4%, and 7.4%, respectively. When the probability threshold was lowered, sensitivity increased. At a threshold of 0.3, for example, Model 1 reached a sensitivity of 42.1% with a specificity of 93.3%, Model 2 reached 43.5% with a specificity of 93.1%, and Model 3 reached 25.2% with a specificity of 95.6%. At a lower threshold of 0.2, the sensitivity for Model 1 increased to 58.7% with a specificity of 87.2%, for Model 2 it reached 62.5% with a specificity of 87.7%, and for Model 3 it reached 45.4% with a specificity of 88%. Calibration plots demonstrated close agreement between predicted and observed risks, consistent with calibration slopes near one and indicating good model calibration (Supplementary Figure S1).

Internal validation using bootstrap resampling with optimism correction proved minimal overfitting across all three models. The performance metric estimates did not differ significantly between the apparent and optimism-corrected performance, indicating good model generalizability. The optimism-corrected Brier scores (0.08) were consistent across the models, ensuring reliable overall prediction accuracy. Calibration slopes remained close to one after correction, suggesting stable calibration. These findings confirm that the models maintained their performance after correcting for internal validation bias (Table 4).

Temporal validation demonstrated preserved discriminative performance across all three models. In the temporally later cohort, test AUC values were comparable to or slightly higher than those observed in the earlier cohort. Model 1 achieved a test AUC of 86.7% compared with a training AUC of 83.8%, Model 2 achieved a test AUC of 87.0% compared with 84.4%, and Model 3 achieved a test AUC of 80.7% compared with 76.3%. The observed differences in the AUC were small, indicating stable model discrimination over time (Table 5).

Decision curve analysis demonstrated that Models 1 and 2 provided greater net benefits than the treat-all and treat-none strategies across a broad range of low-to-moderate threshold probabilities. Model 2 consistently achieved the highest net benefit, while Model 3 showed a lower net benefit across the thresholds. At higher threshold probabilities, the net benefit of all models gradually decreased, indicating that limiting intervention to only those with very-high predicted risk offers little additional advantage. Overall, these findings suggest that the proposed models have potential clinical usefulness within certain decision thresholds (Supplementary Figure S2).

The sensitivity analysis demonstrated that model performance was robust to the representation of fetal biometric predictors. The main model, which was based on centiles, achieved an AUC of 0.847, while the sensitivity model using raw biometric measurements and explicit adjustments for gestational age achieved an AUC of 0.855. The absolute difference in discrimination between the two approaches was minimal. The preservation of model discrimination after the removal of centiles provides strong evidence against circularity and supports that the model captures genuine biological associations related to fetal growth rather than mathematical coupling with the outcome definition (Table 6).

**Figure 2:**
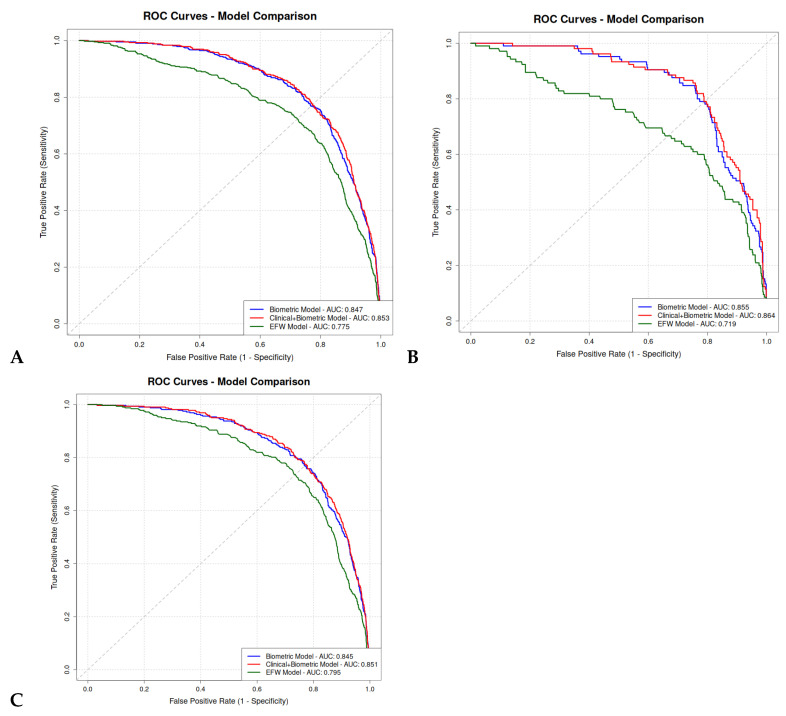
ROC curves comparison of logistic regression prediction models between our subgroups. (**A**) Overall population; (**B**) GDM or pre-existing DM population; (**C**) non-GDM, non-pre-existing DM population.

A total of 726 pregnancies (19%) were complicated by GDM or pre-existing DM. The performance of the logistic regression models did not differ significantly between the two groups (Supplementary Tables S5 and Table S6). The ROC curves of the predictive models are presented in Figure 2.

## 4. Discussion

### 4.1. Primary Findings

The study’s main findings were that (i) maternal conditions, including GDM, pre-existing diabetes, elevated pre-pregnancy BMI, and higher parity, were significantly higher in the LGA group; (ii) sonographic parameters (AC, HC, FL, and mUtA-PI) were significant predictors of an LGA neonate; (iii) the biometric ultrasound measurements model (Model 1) performed similarly to the clinical and biometric model (Model 2), and outperformed the EFW model (Model 3); (iv) all models demonstrated a high degree of flexibility, with an adjustable balance between sensitivity and specificity that can be calibrated by selecting different probability thresholds to suit specific clinical objectives; (v) bootstrap internal validation demonstrated minimal overfitting, with evaluation metrics remaining stable after optimism correction, while temporal validation showed preserved performance across time periods, supporting the robustness of the model; and (vi) decision curve analysis indicated that Models 1 and 2 provided greater net benefit than the treat-all or treat-none strategies across clinically relevant risk thresholds, supporting their potential clinical usefulness.

### 4.2. Interpretation of the Results

Pre-pregnancy BMI, GDM, pre-existing diabetes, and excessive gestational weight gain are recognized risk factors for delivering LGA neonates [16,17,29,30,31]. The underlying pathophysiology involves fetal hyperinsulinemia, an anabolic hormone that promotes accelerated fetal growth and ultimately leads to fetal macrosomia [32]. In addition to metabolic factors, multiparity has consistently been identified as a major risk factor for macrosomia, with studies showing increased odds of LGA with higher parity (aOR 1.31) [33,34,35]. Although a history of PCS has not been extensively studied, existing evidence, including a study by Rosen et al., suggests that PCS is more common among mothers of LGA infants [32]. In our study, women who delivered LGA infants had a higher BMI and were significantly more likely to have GDM or pre-existing diabetes. Both higher parity and PCS were more frequent in the LGA group. Because maternal overweight and obesity are modifiable factors, implementing structured lifestyle and weight-management strategies, particularly among multiparous women, is essential for optimizing maternal and neonatal health outcomes.

Ultrasound-based fetal biometry is widely employed to predict LGA infants, though its diagnostic accuracy is modest; a 2020 meta-analysis demonstrated that sonographically suspected macrosomia had a sensitivity of 53.2% for predicting LGA, a finding consistent across multiple studies [2,36]. It is reasonable that ultrasound appears more effective in predicting LGA infants at 36 weeks compared to 32 weeks; its accuracy increases closer to the delivery date [15]. Fetal measurements in the early third trimester have limited prognostic value [15]. Although a lower HC-to-AC ratio was proposed as a method to increase sensitivity, it failed to improve predictive accuracy in a retrospective study when compared to estimated EFW and AC alone [37]. In our study, we examined a variety of ultrasound measurements to predict the birth of LGA neonates. The most significant individual predictor proved to be AC, with each additional centile associated with 7% higher odds of the neonate being LGA at birth.

The association between mUtA-PI measurements and SGA neonates has been extensively studied, whereas its relationship with LGA outcomes has received less attention [38]. In the study by Ip et al., which examined ultrasonographic parameters at 11 to 13^+6^ gestational weeks, mUtA-PI values were slightly lower in pregnancies that subsequently developed LGA neonates, although the difference was not statistically significant [39]. mUtA-PI proved to be a significant predictor in our study, with each centile increase associated with a 2% reduction in the odds of LGA. While mUtA-PI is traditionally associated with placental insufficiency and SGA, these findings suggest its potential role in predicting LGA, necessitating further research.

The biometric model, incorporating ultrasound and Doppler measurements, achieved strong predictive ability. In an attempt to improve accuracy, we integrated maternal clinical factors into the model. Notably, the combined clinical and biometric model did not outperform the biometric model, suggesting that the inclusion of clinical variables added limited incremental value in our logistic regression models. This finding contrasts with previous studies. Weschenfelder et al. assessed a clinical model and reported improved performance following the addition of ultrasound parameters [40]. Similarly, Erkamp et al. found that the combination of clinical variables with ultrasound and Doppler data improved predictive accuracy compared to clinical data alone [41]. These divergent results highlight the complexity of predicting perinatal outcomes and suggest that the contribution of clinical and ultrasonographic variables may vary depending on the study population, timing of assessment, and model structure.

The biometric model demonstrated superior predictive performance compared to the EFW model, with AUCs of 84.7% and 77.5%, respectively. This result may be attributed to the model’s assessment of all fetal biometric measurements individually rather than the EFW alone, but also to the addition of Doppler ultrasound measurements and amniotic fluid assessment. In the literature, Pilalis et al. have reported a similar comparison, showing that incorporating Doppler and clinical variables into an EFW model significantly improved predictive accuracy [42]. These results reveal the limitations of generalized predictive formulas, suggesting that population-specific modeling may offer superior performance. However, the direct comparison of EFW to its individual components plus Doppler may not be methodologically neutral and could disadvantage the EFW model. Future work should test models that integrate EFW with Doppler and maternal variables. Moreover, given the heterogeneity in maternal and fetal characteristics across populations, clinical centers may benefit from developing models based on individual measurements tailored to their specific population, thereby optimizing predictive ability.

### 4.3. Strengths and Limitations

This study has several notable strengths. First, we comprehensively assessed multiple fetal biometric measurements individually, including HC, AC, FL, and HC-to-AC ratio, which proved to be superior in predicting LGA neonates compared to the EFW. Notably, the study also uniquely incorporated mUtA-PI and the presence of polyhydramnios, parameters not typically assessed in similar studies, offering a more complete picture of fetal and placental hemodynamics. Moreover, this population was routinely screened in the first trimester, all pregnancies were appropriately dated, and all scans were performed by trained physicians. Also, all women underwent routine GDM screening at 26^+0^ to 27^+6^ weeks. Another major advantage is the internal validation of the models using bootstrap resampling, with evaluation metrics remaining stable before and after optimism correction, and preserved performance on temporal validation, indicating reliable, well-calibrated, and generalizable predictions. An additional strength of this study is the use of decision curve analysis, which demonstrated that Models 1 and 2 provide greater net benefits than the treat-all or treat-none strategies across clinically relevant risk thresholds, supporting their potential clinical usefulness. Finally, the development of three distinct multivariable prediction models, together with subgroup analyses for pregnancies with and without GDM or pre-existing diabetes, provides valuable information on model performance across different clinical contexts.

Several limitations should also be acknowledged. First, the retrospective, single-center design may limit the generalizability of the findings to more diverse populations. External validation in independent cohorts is therefore essential and is planned as the next step. Although decision curve analysis suggested potential clinical usefulness, the models still need to be evaluated in clinical settings. Prospective validation is required to determine whether using these models improves maternal and neonatal outcomes and whether they can be integrated into routine third-trimester care. Second, the assessment window of 30–34 weeks was selected to align with the standard timing of the third-trimester ultrasound scan in Greece, which enhances clinical applicability but restricts the evaluation of data obtained beyond 34 weeks, when substantial fetal growth occurs. A third consideration is the models’ performance at the conventional 0.5 probability threshold, which yields high specificity at the cost of low sensitivity. Rather than an inherent model weakness, this reflects a specific operating point that prioritizes minimizing false-positive results. The models’ clinical value lies in their flexibility; by adjusting the decision threshold, sensitivity can be substantially increased to approximately 80% (at a 0.1 threshold), allowing the tool to be calibrated for different clinical objectives. Finally, other limitations include the imbalanced size of the LGA and non-LGA groups and the unavailability of data on a prior history of delivering an LGA neonate, which is a known risk factor.

## 5. Conclusions

In summary, this study showed that a prediction model based on individual fetal biometric and Doppler measurements at 30–34 weeks can predict LGA neonates at birth with good accuracy, outperforming a model based solely on EFW and performing similarly to a model that additionally includes maternal factors. The model demonstrated adequate discriminative ability, with an AUC of up to 84.8%, and important clinical flexibility. By adjusting the decision threshold, sensitivity can be increased from around 23.4% to over 58% while maintaining high specificity (>87%). This adaptability, driven by threshold selection, establishes the model as a valuable and practical tool for early LGA risk stratification. Following internal bootstrap validation, temporal validation, and decision curve analysis demonstrating robust performance and potential clinical utility, external validation in independent populations represents the essential next step before clinical implementation.

## Figures and Tables

**Table 1:** Maternal characteristics and measured variables between the LGA and non-LGA groups.

Characteristics	Non-LGA (3377)	LGA (431)	*p*-Value
AC centile	35.9 (23.8,50.5)	62.5 (50.3,76.4)	<0.0001
HC centile	32.2 (18.0,50.1)	53.7 (38.8,71.3)	<0.0001
FL centile	36.3 (20.0,55.7)	56.2 (37.3,74.2)	<0.0001
HC/AC centile	29.81 (3.35,77.54)	3.09 (0.084,29.25)	<0.0001
mUtA-PI centile	56.3 (41.7,73.5)	50.3 (36.7,63.4)	<0.0001
Polyhydramnios	2 (0.059%)	5 (1.16%)	<0.0001
Pre-pregnancy BMI	22.7 (20.7,26.0)	24.1 (21.8,27.7)	<0.0001
Maternal Age	32.57 (±5.2)	32.58 (±5.4)	0.98
Bilateral UtA notch	20 (0.5%)	0 (0%)	-
Left UtA notch	49 (1.4%)	1 (0.2%)	0.062
Right UtA notch	38 (1.1%)	1 (0.2%)	0.14
No UtA notch	3270 (96.8%)	429 (99.5%)	0.003
PCS	432 (12.7%)	83 (19.2%)	<0.0001
GDM	613 (18.1%)	100 (23.2%)	0.014
ART	238 (7.0%)	32 (7.4%)	0.85
Multiparity	1243 (36.8%)	193 (44.7%)	0.002
Pre-existing DM	10 (0.2%)	10 (2.3%)	<0.0001
Smoking	383 (11.3%)	47 (10.9%)	0.85
Hypothyroidism	415 (12.2%)	65 (15.0%)	0.12
Chronic hypertension	35 (1.0%)	2 (0.4%)	0.38

mUtA-PI: mean artery pulsatility index; PCS: previous cesarean section; GDM: gestational diabetes mellitus; ART: conception with the use of assisted reproductive technology; pre-existing DM is defined as pre-existing type 1 or 2 diabetes mellitus; BMI: body mass index; AC: abdominal circumference; HC: head circumference; FL: femur length. Continuous variables are reported as median and interquartile range, while categorical variables are reported as number and percentage.

**Table 2:** Multivariate adjusted ORs for ultrasound and Doppler variables.

Measured Variables	aOR (95% CI)	*p*-Value
AC centile	1.07 (1.06,1.08)	<0.0001
HC/AC centile	1.01 (1.006,1.01)	<0.0001
FL centile	1.01 (1.009,1.01)	<0.0001
mUtA-PI centile	0.98 (0.98,0.99)	<0.0001
Polyhydramnios	4.97 (0.7,58.8)	0.14

AC: abdominal circumference; aOR: adjusted odds ratio; HC: head circumference; FL: femur length; mUtA-PI: mean artery pulsatility index.

**Table 3:** Predictive performance comparison of the prediction models at the conventional 0.5 threshold.

	Model 1	Model2	Model 3
Sensitivity	23.4 [19.6,27.6]	23.4 [19.5,27.6]	7.4 [5.3,10.3]
Specificity	98.2 [97.7,98.6]	98.2 [97.7,98.6]	99.2 [98.8,99.4]
PPV	64.1 [56.3,71.2]	63.6 [55.9,70.8]	55.1 [42.4,67.2]
NPV	90.8 [89.8,91.7]	90.8 [89.8,91.7]	89.2 [88.1,90.1]
AUC	84.7 [8.9,86.5]	85.3 [83.5,87.1]	77.5 [75.1,79.9]
Post hoc power analysis	1	1	1
Systematic error of ROC curve	4.04 × 10^−15^	8.08 × 10^−15^	−1.07 × 10^−15^
Calibration slope	1	1	1

Model 1: biometric ultrasound measurements model; Model 2: biometric and clinical model; Model 3: EFW model; PPV: positive predictive value; NPV: negative predictive value; AUC: area under the curve; prognostic measures are reported as values (%) [95% confidence intervals].

**Table 4:** Internal validation of the prediction models.

	Model 1			Model 2			Model 3		
	Apparent	Optimism	Corrected	Apparent	Optimism	Corrected	Apparent	Optimism	Corrected
AUC	84.7	0.06	84.6	85.3	0.5	84.8	77.5	0.03	77.5
Brier Score	0.08	−0.0003	0.08	0.07	−0.001	0.08	0.08	−0.0001	0.08
Calibration Slope	1	0.01	0.98	1	0.03	0.96	1	−0.0023	1.002
Sensitivity	23.4	0.1	23.2	23.4	0.7	22.7	7.4	0.04	7.4
Specificity	98.2	0.02	98.2	98.2	0.06	98.1	99.2	0.001	99.2
PPV	64.1	0.3	63.6	61.1	1.4	62.2	55.1	−0.4	55.5
NPV	90.8	0.005	90.8	90.8	0.08	90.7	89.2	0.009	89.1

Model 1: biometric ultrasound measurements model; Model 2: biometric and clinical model; Model 3: EFW model; Apparent: performance on the training data; Optimism: average overestimation of performance from bootstrap validation; Optimism-corrected: obtained by subtracting the estimated optimism from the apparent performance, thereby reflecting the model’s expected performance when applied to new data; AUC: area under the curve; PPV: positive predictive value; NPV: negative predictive value; prognostic measures are reported as values (%) and were calculated at the conventional 0.5 probability threshold.

**Table 5:** Temporal validation of the prediction models.

	Training *n* (LGA)	Test *n* (LGA)	Training AUC (%)	Test AUC (%)	Difference AUC (%)
Model 1	2599 (291)	1118 (136)	83.8	86.7 (83.7,89.6)	−2.9
Model 2	2599 (291)	1118 (136)	84.4	87 (84.3,89.8)	−2.6
Model 3	2599 (291)	1118 (136)	76.3	80.7 (76.7,84.7)	−4.4

Model 1: biometric ultrasound measurements model; Model 2: biometric and clinical model; Model 3: EFW model.

**Table 6:** Circularity sensitivity analysis.

Analysis Approach	Representation of Fetal Biometry	Included Variables	AUC (%)
Main model	Gestational age-adjusted centiles	AC centile, HC/AC centile, FL centile, mUtA-PI centile, and polyhydramnios.	84.7
Sensitivity model	Raw measurements	AC (mm), HC (mm), FL (mm), gestational age (days), mUtA-PI (raw), and polyhydramnios.	85.5
Absolute difference			0.7

AC: abdominal circumference; HC: head circumference; FL: femur length; mUtA-PI: mean uterine artery pulsatility index; AUC: area under the curve; mm: millimiters.

## Data Availability

The data presented in this study are available on reasonable request from the corresponding author. The data are not publicly available due to privacy restrictions.

## Supplementary Materials

The following supporting information can be downloaded at: https://www.mdpi.com/article/10.3390/diagnostics16020187/s1, Figure S1: Calibration plots of prediction models; Figure S2: Decision curve analysis; Table S1: Missing data table; Table S2: Predictive performance of prediction model 1 calculated for various probability thresholds; Table S3: Predictive performance of prediction model 2 calculated for various probability thresholds; Table S4: Predictive performance of prediction model 3 calculated for various probability thresholds; Table S5: Predictive performance comparison of the prediction models of GDM or pre-existing diabetes’ population; Table S6: Predictive performance comparison of the prediction models of non-GDM, non-pre-existing diabetes’ population.
