# Supplementary material for: Predictive Accuracy of Ultrasound Biometry and Maternal Factors in Identifying Large-for-Gestational-Age Neonates at 30–34 Weeks
Source: Diagnostics (Basel). 2026 Jan 7;16(2):187. doi: 10.3390/diagnostics16020187 (PMC12839821; doi:10.3390/diagnostics16020187)

diagnostics

MDPI

Supplementary Tables

Supplementary Table S1. Missing data table

Variable	Missing	Total	Percent (%)
Abdominal circumference (AC)	0	3808	0.0
Head circumference (HC)	0	3808	0.0
Femur length (FL)	0	3808	0.0
HC to AC	0	3808	0.0
Mean uterine artery PI	0	3808	0.0
Polyhydramnios	0	3808	0.0
Maternal age	0	3808	0.0
Pre-pregnancy BMI	89	3808	2.34
Parity	0	3808	0.0
Gestational diabetes mellitus	0	3808	0.0
Pre-existing diabetes Mellitus	0	3808	0.0
Previous cesarean section	0	3808	0.0
Assisted reproduction technologies use	0	3808	0.0
Smoking	0	3808	0.0
Hypothyroism	0	3808	0.0
Chronic Hypertension	0	3808	0.0
Estimated fetal weight	0	3808	0.0

Supplementary Table S2. Predictive performance of prediction model 1 calculated for various probability thresholds.

	Model 1								
Threshold	0.1	0.2	0.3	0.4	0.5	0.6	0.7	0.8	0.9
Sensitivity	81.7	58.7	42.1	30.4	23.4	12.6	7.2	2.3	0.7
Specificity	73.3	87.2	93.3	96.2	98.2	99.2	99.7	99.8	100
PPV	28.4	37.5	45.2	51.5	64.1	68.3	77.5	71.4	100
NPV	96.8	94.2	92.5	91.4	90.8	89.7	89.2	88.7	88.5

Model 1: biometric ultrasound measurements model; threshold: the probability at which we decide to label the prediction as positive; PPV: postive predictive value; NPV: negative predictive value; prognostic measures are reported as value (%)

Supplementary Table S3. Predictive performance of prediction model 2 calculated for various probability thresholds.

	Model 2								
Threshold	0.1	0.2	0.3	0.4	0.5	0.6	0.7	0.8	0.9
Sensitivity	81.2	62.5	43.5	32.5	23.4	14	8.1	4.2	0.9
Specificity	73.7	87.7	93.1	96.2	98.2	99	99.5	99.8	100
PPV	28.7	39.9	45.3	52.8	63.9	65.9	70	81.8	100
NPV	96.8	94.7	92.7	91.6	90.8	89.8	89.3	88.9	88.6

Model 2: biometric and clinical model; threshold: the probability at which we decide to label the prediction as positive;PPV: positive predictive value; NPV: negative predictive value; prognostic measures are reported as value (%)

Supplementary Table S4. Predictive performance of prediction model 3 calculated for various probability thresholds.

	Model 3								
Threshold	0.1	0.2	0.3	0.4	0.5	0.6	0.7	0.8	0.9
Sensitivity	77.7	45.4	25.2	14.7	7.4	4.2	2.5	1.6	0.7
Specificity	64.2	88	95.6	98.2	99.2	99.6	99.9	99.9	100
PPV	22	33.1	42.8	52.9	55.1	64.2	78.5	87.5	100
NPV	95.6	92.5	90.7	89.8	89.2	88.9	88.7	88.6	88.5

Model 3: EFW model; threshold: the probability at which we decide to label the prediction as positive;PPV: positive predictive value; NPV: negative predictive value; prognostic measures are reported as value (%)

Supplementary Table S5. Predictive performance comparison of the prediction models of GDM or pre-existing diabetes' population

	Model 1	Model2	Model3
Sensitivity	31.4 [23.3, 40.8]	34.2 [25.9, 43.7]	8.5 [4.5, 15.4]
Specificity	97.4 [95.8,98.4]	97.8[96.4, 98.7]	99.5 [98.5, 99.8]
PPV	67.3 [53.3, 78.7]	73.4 [59.7, 83.7]	75 [46.7, 91.1]
NPV	89.3 [86.7, 91.4]	89.7 [87.2, 91.8]	86.4 [83.7, 88.8]
AUC	85.4 [81.8, 89]	86.3 [82.8, 89.8]	71.8 [65.9, 77.7]
Post hoc power analysis	1	1	1

Systematic error of ROC curve	8.82*10^-13	4.16*10^-13	1.33*10^-12

Model 1: biometric ultrasound measurements model; Model 2: biometric and clinical model; Model 3: EFW model; GDM: gestational diabetes mellitus; PPV: positive predictive value; NPV: negative predictive value; AUC: area under the curve; prognostic measures are reported as value (%) [95% confidence intervals]

Supplementary Table S6. Predictive performance comparison of the prediction models of non-GDM, non-pre-existing diabetes' population

	**Model 1**	**Model 2**	**Model 3**
Sensitivity	19.8 [15.8, 24.5]	20.1 [16.1, 24.9]	7.4 [5, 10.8]
Specificity	98.5 [97.9, 98.8]	98.4 [97.9, 98.8]	99 [98.5, 99.3]
PPV	61.5 [51.9, 70.3]	61.3 [51.8, 70]	48 [34.7, 61.4]
NPV	91 [89.9, 92]	91.1 [90, 92]	89.8 [88.7, 90.9]
AUC	84.5 [82.4, 86.6]	85.1 [83, 87.1]	79.4 [76.9, 81.9]
Post hoc power analysis	1	1	1
Systematic error of ROC curve	4.02*10^-13	5.93*10^-13	5.41*10^-15

Model 1: biometric ultrasound measurements model; Model 2: biometric and clinical model; Model 3: EFW model; GDM: gestational diabetes mellitus; PPV: positive predictive value; NPV: negative predictive value; AUC: area under the curve; prognostic measures are reported as value (%) [95% confidence intervals]

Supplementary Figure S1. Calibration plots of prediction models

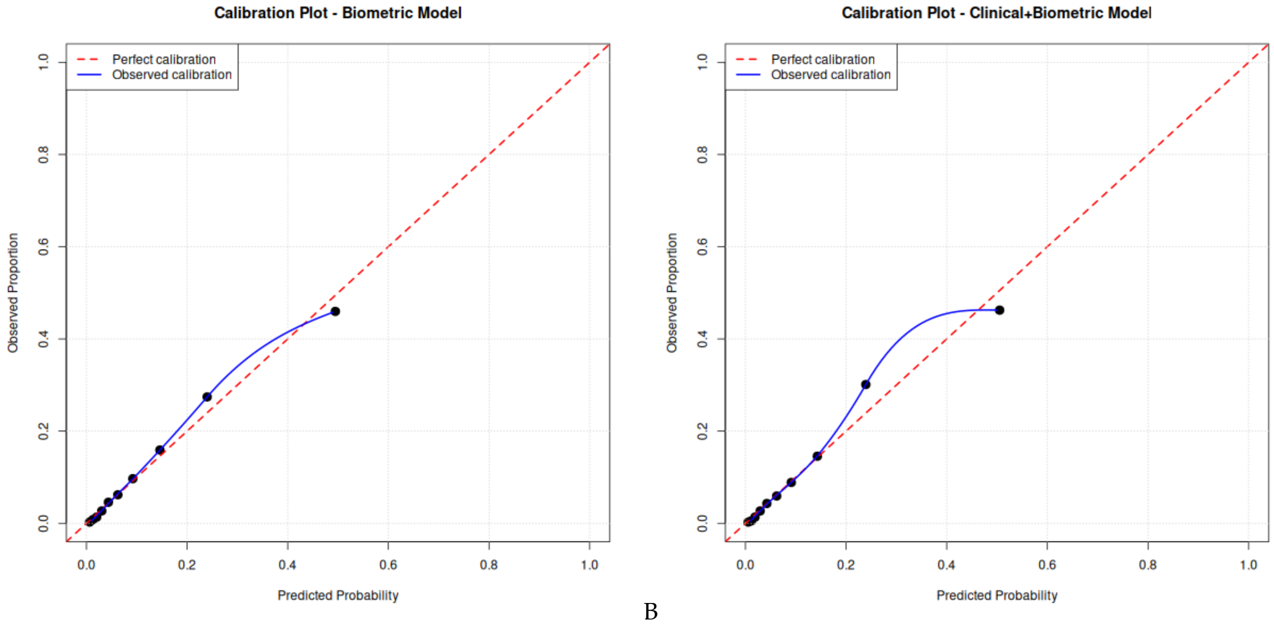

A B

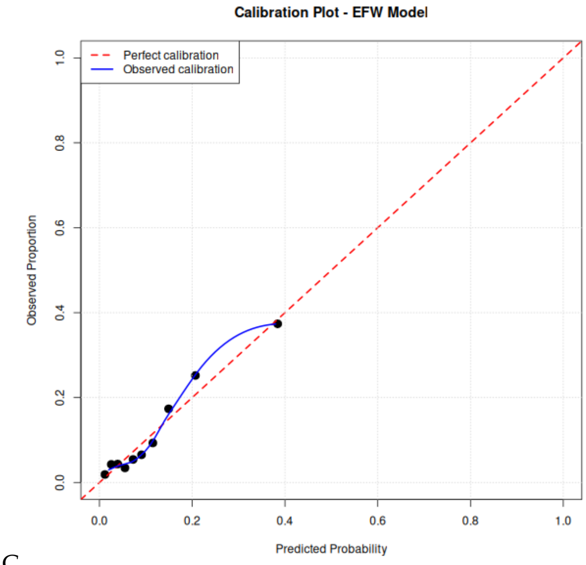

C

A: Calibration plot of Model 1 / biometric ultrasound measurements model

B: Calibration plot of Model 2 / biometric and clinical model

C: Calibration plot of Model 3 / EFW model

Supplementary Figure S2. Decision curve analysis

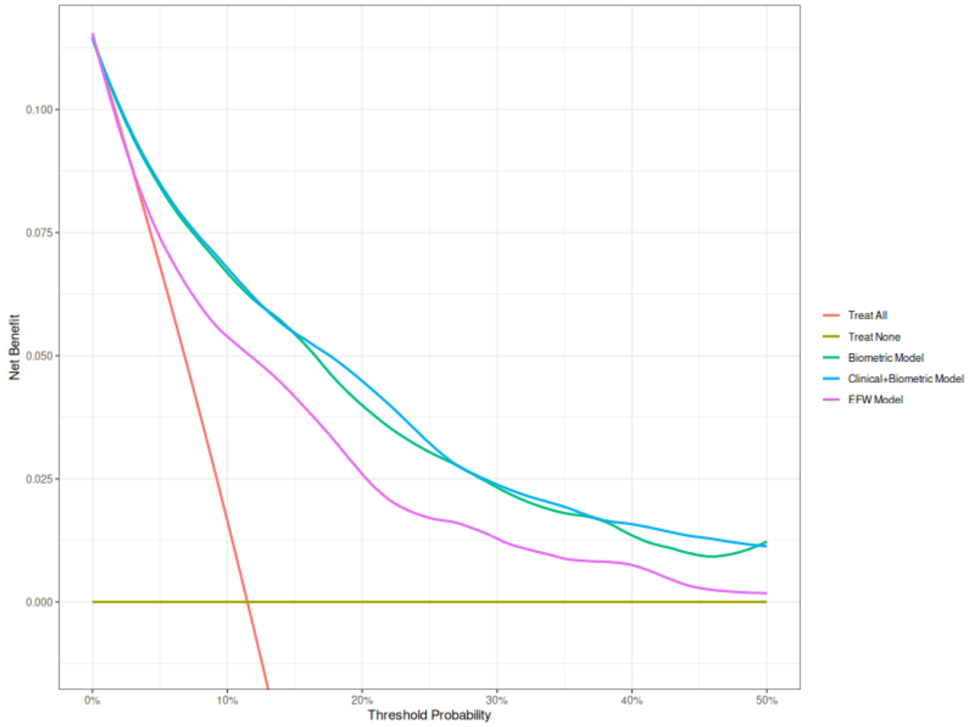

Supplement: Supplementary file 1 [file diagnostics-16-00187-s001.zip › diagnostics-3990852-supplementary.pdf]
